# Prevalence of and prevention for work-related upper limb disorders among physical therapists: a systematic review

**DOI:** 10.1186/s12891-022-05412-8

**Published:** 2022-05-14

**Authors:** Eliza Waller, Andrea Bowens, Nicholas Washmuth

**Affiliations:** Department of Physical Therapy, Samford University, 800 Lakeshore Drive, Birmingham, AL 35229 USA

**Keywords:** Work-related upper limb disorders, Physical therapists, Prevalence, Risk factors, Prevention

## Abstract

**Background:**

Physical therapists (PTs) are at increased risk for development of work-related upper limb disorders (WRULDs) due to the physically intensive, constant hands-on nature of the profession. The objectives of this systematic review were to examine the literature on WRULDs among PTs, specifically the (1) 1-year prevalence, (2) workplace risk factors, (3) consequences, and (4) coping strategies utilized to mitigate WRULDs.

**Methods:**

A comprehensive search of the literature was performed using PubMed, CINHAL, EMBASE, and Google Scholar. The Preferred Reporting Items for Systematic Reviews and Meta-Analyses (PRISMA) guidelines were used for conducting this systematic review. Studies that reported the 1-year prevalence of WRULDs among PTs, workplace risk factors for WRULDs, consequences of WRULDs, and coping strategies utilized by PTs were included.

**Results:**

Twelve studies met the inclusion criteria. The 1-year WRULDs prevalence rates varied widely, with thumb disorders having the highest prevalence (7.6-52.5%), followed by wrist and hand disorders (5-66.2%), shoulder disorders (3.2-45.2%), and elbow disorders (4-16%). Reported risk factors included treating a high volume of patients and frequent performance of manual therapy techniques. Consequences included interference with PTs’ personal and professional activities while coping strategies involved alterations to the work environment, techniques used, and workload.

**Conclusions:**

WRULDs remain a persistent threat to the PT workforce, likely due to the hands-on, physically intensive nature of professional activities. An essential strategy to reduce WRULDs is to improve clinicians’ awareness of WRULDs, workplace risk factors, and subsequent consequences of WRULDs. Effective coping strategies are critical to preserve, protect, and prolong PTs’ use of the upper limbs.

## Introduction

Work-related musculoskeletal disorders (WMSDs) continue to rise among health care professionals and this rise is accompanied by spiraling costs across the healthcare delivery system. For instance, in 2014 WMSDs accounted for 5.8% of the United States’ gross domestic product, posing a significant economic burden to workers and their families, employers, and society due to the loss of income and productivity, increased medical expenses and workers’ compensation claims, and Social Security disability payments [1].

The US Department of Labor defines WMSDs as injuries or disorders of the muscles, nerves, tendons, joints, cartilage, and the multiple elements of the spinal column [2]. WMSDs of these structures clinically manifest as sprains, strains, soreness, tears, joint pain, inflammation, and altered joint mobility [3]. These disorders affect all healthcare professions, especially physically demanding occupations such as dentists, nurses, surgeons, occupational and physical therapists [4].

Physical therapists (PTs), specifically, are at an increased risk for developing work-related upper limb disorders (WRULDs) due to the physically intensive and constant hands-on nature of the profession [5]. WRULDs involve WMSDs of the neck, shoulders, elbows, forearms, wrists, and hands, and most commonly result from the generated forces and repetitive tasks required of the profession [6]. Historically, disorders of the low back have been the subject of greatest research and education in the physical therapy profession [7]. This disproportionate focus may be related to underreporting of WRULDs and the stoic culture of physical therapy [8, 9].

Therefore, the purpose of this systematic review was to examine the literature regarding WRULDs, specifically in the following four areas: 1) the 1-year prevalence of WRULDs among physical therapists (PTs), 2) the work-relevant risk factors in the physical therapy workplace that may exacerbate the development of WRULDs, 3) the consequences of WRULDs, and 4) the coping strategies utilized for prevention and intervention purposes to mitigate WRULDs among PTs.

## Methods

The Preferred Reporting Items for Systematic Reviews and Meta-Analyses (PRISMA) guidelines [10] were used for this systematic review.

### Literature search and data management

A systematic search of the literature was performed using PubMed, CINHAL, EMBASE, and Google Scholar databases to identify pertinent, peer-reviewed journal articles that focused on the topic of WRULDs among PTs and met the established inclusion and exclusion criteria. The search terms used were various combinations of key words and phrases including work-related musculoskeletal disorders, upper limb disorders, occupational injuries, physical therapists, prevalence, incidence, risk factors, and prevention. The following Medical Subject Headings (MeSH) terms were used to search the databases:

- “Healthcare workers AND work-related AND (musculoskeletal disorders OR injuries OR pain)”
- “Work-related AND (musculoskeletal disorders OR injuries OR pain) AND (physical therapists OR physiotherapists)”
- “(Occupational disorders OR injuries) AND (physical therapists OR physiotherapists)”
- “(Upper limb disorders OR upper extremity disorders) AND (physical therapists OR physiotherapists)”
- “(Upper limb disorders OR upper extremity disorders) AND (prevalence OR incidence) AND (physical therapists OR physiotherapists)”
- “Prevention AND Work-Related AND (musculoskeletal disorders OR injuries OR pain)”
- “Prevention AND Work-Related AND (musculoskeletal disorders OR injuries OR pain) AND (physical therapists OR physiotherapists)”

### Inclusion and exclusion criteria

The 211 potentially eligible full-text articles were further screened according to specific inclusion and exclusion criteria. For inclusion in the systematic review, it was required that articles be peer-reviewed, published between January 2000 and March 2022, published in the English language, available in full-text, and included study designs of qualitative and/or quantitative methodology. Only studies that reported WMSD(s) involving the shoulders, elbows, wrists, and/or hands among physical therapists were included. Cervical spine WMSDs were not included due to widespread inconsistency in the literature as to whether neck WMSDs fell under the spine category or the upper extremity category [11]. Articles that focused solely on the prevalence of work-related non-upper limb disorders, such as low back pain, among PTs as well as articles that included professions other than PTs were excluded from this systematic review. For articles to be included, they had to report the 1-year prevalence of WMSDs of the upper extremity (≥1 body region) among PTs, cite workplace risk factors for the development of WRULDs, mention consequences of and coping strategies for WRULDs.

### Study selection

**Fig. 1:**
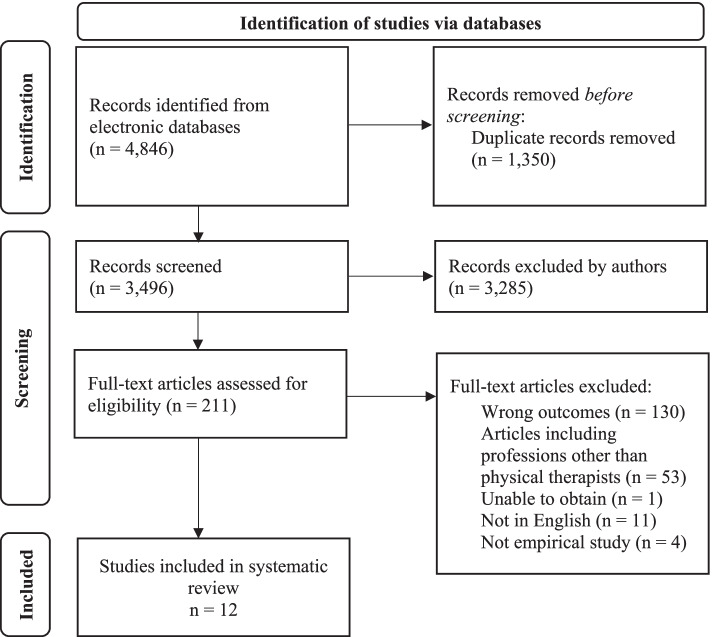
PRISMA Flow Diagram

As displayed in Fig. 1, the initial search resulted in 4,846 articles. After removing duplicate records, 3,496 journal articles remained. Two reviewers (E.W. and A.B.) screened the article titles and abstracts using the established criteria, which resulted in the exclusion of 3,285 articles that were unrelated to the research question. The remaining 211 full-text journal articles were obtained and reviewed by two authors (E.W. and A.B.) for eligibility. Any discrepancies in the inclusion or exclusion of specific articles were discussed and resolved by a third reviewer (N.W.). This resulted in 12 total articles that met all criteria and included in this review.

### Risk of bias assessment

The Joanna Briggs Institute (JBI) Critical Appraisal Checklist for Studies Reporting Prevalence Data [12] was used to systematically assess the risk of bias in the 12 studies. Based on its methodological assessment of the risk of bias in prevalence studies and its detailed descriptions of how to rate each criterion, this tool was deemed most suitable for this systematic review. The JBI checklist tool [12] assessed the risk of bias by considering nine criteria (Appendix): representative target population, appropriate participant recruitment, adequate sample size, subject and setting description, data analysis coverage, measurement of outcome, measurement reliability, appropriate statistical analysis, and adequate response rate.

The JBI checklist tool [12] was applied to the 12 identified studies and the nine criteria were recorded as either “yes, no, unclear, or not applicable” according to the tool’s extensive descriptions. For each criterion recorded as “yes” it received a score of 1 and for each criterion recorded as “no” it received a score of 0, with a total score ranging from 0-9. Studies with >70% of “yes” ratings were considered low risk of bias, studies between 50% and 69% of “yes” ratings were considered moderate risk of bias, and studies with “yes” ratings <50% were considered high risk of bias.

### Data extraction and analysis

Table 1 displays the characteristics of each article in this systematic review, including the year of publication, place of study, study design, number of participants, and outcome measures of each study.

**Table 1:** Study Characteristics

**Author**	**Year of Publication**	**Place of Study**	**Study Design**	**Number of Participants (n)**	**Outcome Measure**
Adegoke et al. [13]	2008	Nigeria	Cross-Sectional Study	126 (46F, 80M)	Survey based on Cromie et al. [5] and West and Gardner [21]
Alnaser and Aljadi [14]	2019	State of Kuwait	Descriptive Cross-Sectional Study	312 (186F, 126M)	Questionnaire adapted from Holder et al. [15]
Buddhadev and Kotecha [16]	2012	Saurashtra Region, India	Cross-Sectional Study	29^a^	Self-designed questionnaire
Campo et al. [17]	2019	United States	Mixed-Methods Study	962 (576F, 382M)	Self-designed survey based on Campo et al. [16] and the NMQ [22]
Campo et al. [18]	2008	United States	Prospective Cohort Study w/ 1-year Follow-Up	881 (627F, 254M)	Self-designed questionnaire based on the NMQ [22]
Chung et al. [19]	2013	Korea	Cross-Sectional Study	157 (74F, 83M)	Self-administered questionnaire based on Adegoke et al. [13]
Cromie et al. [5]	2000	Australia	Cross-Sectional Study	536 (418F, 118M)	Self-designed survey WMSD aspect based on the NMQ [22]
Glover et al. [20]	2005	United Kindom	Cross-Sectional Study	2593 (2318F, 275M)	Self-designed survey based on Cromie et al.[5], West and Gardner [24], and the Standardised Nordic Questionnaire [25]
McMahon et al. [21]	2006	Australia	Cross-Sectional Study	961 (746F, 215M)	Self-designed questionnaire
Rossettini et al. [22]	2016	Italy	Cross-Sectional Study	219 (90F, 126M)	Self-designed questionnaire
Rozenfeld et al. [23]	2010	Israel	Cross-Sectional Study	123 (82F, 41M)	Questionnaire based on Cromie et al. [5]
West and Gardner [24]	2001	Australia	Cross-Sectional Study	217 (178F, 39M)	Self-designed questionnaire

*Abbreviations*: *F* Female, *M* Male

^a^Gender breakdown not reported

## Results

Of the 211 articles examined for inclusion, 12 studies [5, 13, 14, 16–24] met the criteria for this systematic review, including 11 observational studies and one mixed-methods study (Table 1). These studies were conducted in numerous geographical locations including Nigeria, India, United States of America, United Kingdom, Korea, Australia, Italy, Kuwait, and Israel. Sample sizes ranged from 29 PTs to 2,593 PTs, with a majority of these studies having a higher female to male ratio [5, 13, 14, 17, 18, 21, 23, 24].

**Fig. 2:**
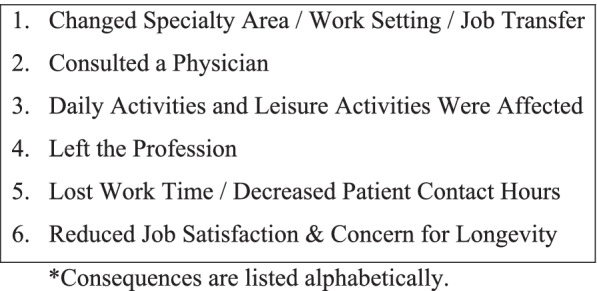
Consequences of WRULDs*

**Fig. 3:**
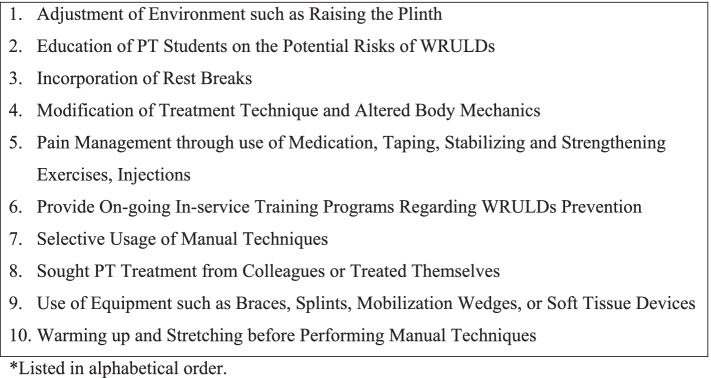
Coping Strategies, Treatment of and Prevention for WRULDs*

The following information was extracted from the 12 eligible full-text articles and organized into a table: 1-year prevalence of general WMSDs, the career prevalence of WMSDs (if reported), and the 1-year prevalence of WRULDs reported by body region (Table 3). Other data extracted from the articles included PT-identified workplace risk factors (Table 4), consequences of WRULDs (Fig. 2), and coping strategies for WRULDs (Fig. 3).

### Risk of bias assessment

The JBI Critical Appraisal Checklist for Prevalence Studies [12] was used to assess the risk of bias for each of the included studies. Table 2 displays the assessment of the nine criteria and the corresponding risk of bias (low, moderate, or high) for each study that were agreed upon by two reviewers. Eleven of the 12 studies [5, 13, 14, 17–24] were characterized as having a low risk of bias, while one study [16] was deemed to have a moderate risk of bias. All studies had representative target populations, appropriate participant recruitment, sufficient data analysis coverage, and proper measurement reliability. The three most common unmet criteria were failure to use valid methods for identifying the condition, failure to use appropriate statistical analysis, and inadequate response rates.

**Table 2:** Risk of bias assessment of the 10 included studies

**Study**	**Criteria and Corresponding Scores**^a^										
	**Q1**	**Q2**	**Q3**	**Q4**	**Q5**	**Q6**	**Q7**	**Q8**	**Q9**	**%**	**Risk of Bias**
Adegoke et al. [13]	1	1	1	1	1	1	1	1	1	100%	Low
Alnaser and Aljadi [14]	1	1	0	1	1	1	1	0	1	78%	Low
Buddhadev and Kotecha [16]	1	1	0	1	1	0	1	0	1	67%	Moderate
Campo et al. [17]	1	1	1	1	1	1	1	1	0	89%	Low
Campo et al. [18]	1	1	1	1	1	1	1	1	1	100%	Low
Chung et al. [19]	1	1	1	0	1	1	1	1	1	89%	Low
Cromie et al. [5]	1	1	1	1	1	1	1	1	1	100%	Low
Glover et al. [20]	1	1	1	1	1	1	1	1	1	100%	Low
McMahon et al. [21]	1	1	1	1	1	0	1	1	1	89%	Low
Rossettini et al. [22]	1	1	1	1	1	1	1	1	0	89%	Low
Rozenfeld et al. [23]	1	1	1	1	1	1	1	1	1	100%	Low
West and Gardner [24]	1	1	1	1	1	1	1	0	1	89%	Low

^a^Q1-Q9 indicate questions 1 to 9 based on the JBI criteria located in Appendix

Score of 1: indicates the article does fulfill the specified criteria

Score of 0: indicates the article does not fulfill the stated criteria

Studies with >70% = low risk of bias, studies with 50-69% = moderate risk of bias, and studies with <50% = high risk of bias

### Outcome measures

All 12 studies included in this review utilized some form of questionnaire or survey, either an author-designed questionnaire, a survey based on another study’s questionnaire, or a standardized questionnaire such as the Nordic Musculoskeletal Questionnaire (NMQ) [25]. These questionnaires served as a subjective measurement of the frequency of PTs’ musculoskeletal symptoms and the anatomic region(s) affected. Other components of these questionnaires assessed the PT-identified risk factors for and consequences of WMSDs, as well as the coping strategies or preventative measures employed by the PTs in response to their WMSDs.

### Prevalence of WRULDs among PTs according to body region

The 1-year prevalence of general WMSDs across all body regions among PTs ranged from 28% to 92.4% and the career prevalence of general WMSDs ranged from 40% to 91% [5, 20–24]. The reported 1-year prevalence of WRULDs and the affected body region percentages are presented in Table 3. Specifically, the 1-year prevalence of shoulder disorders among PTs, reported by nine of the 12 studies [5, 13, 14, 16, 18–20, 23, 24], ranged between 10% to 45.2%. Only seven studies [5, 13, 16, 18–20, 23] stated the 1-year prevalence of the elbow/forearm region with values between 1.4% and 16%. The 1-year prevalence of wrist and hand disorders varied broadly among the 10 reporting studies [5, 13, 14, 16–20, 23, 24] with 5% to 66.2% of PTs reporting wrist and hand disorders. It should be noted that in four of the 12 studies [14, 16, 18, 24] thumb disorders were not separated from wrist and hand data, whereas the remaining eight studies [5, 13, 17, 19–23] considered the thumb region separately. In these eight studies, thumb disorders were frequently reported by PTs across studies, with 1-year prevalence ranging from 7.6% to 52.5%. Campo et al. [17] reported a combined 1-year prevalence of wrist, hand, and thumb disorders of 74.8% and prevalence of isolated thumb disorders of 52.5% among a sample of exclusively orthopedic PTs. Two other studies also identified a high prevalence of thumb disorders among physical therapists (>40%) [21, 22]. In contrast, Chung [19] reported 1-year prevalence of work-related thumb disorders as only 7.6%.

**Table 3:** Prevalence of WMSDs and Prevalence of WRULDs by Body Region

**Author**	**1 **^**-**^**Year Prevalence of WMSDs (%)**	**1**^**-**^**Year Prevalence of WRULDs** **By Body Region (%)**
Adegoke et al. [13]	91.3%	Shoulder: 22.2%
		Elbow/Forearm: 5.6%
		Wrist & Hand: 20.6%
		Thumb: 11.1%
Alnaser and Aljadi [14]	48%	Shoulder: 7%
		Elbow/Forearm: ^a^
		Wrist & Hand: 20%
		Thumb: ^ab^
Buddhadev and Kotecha [16]	69%	Shoulder: 15%
		Elbow/Forearm: 5%
		Wrist & Hand: 5%
		Thumb: ^ab^
Campo et al. [17]	74.8% (1-Year Prevalence of wrist, hand, and thumb WMSDs)	Shoulder: ^a^
		Elbow/Forearm: ^a^
		Wrist & Hand: 66.2%
		Thumb: 52.5%
Campo et al. [18]	28%	Shoulder: 3.2%
		Elbow/Forearm: 1.4%
		Wrist & Hand: 5.3%
		Thumb: ^ab^
Chung et al. [19]	92.4%	Shoulder: 45.2%
		Elbow/Forearm: 7.0%
		Wrist & Hand: 33.8%
		Thumb: 7.6%
Cromie et al. [5]	82.8% Career Prevalence of 91%	Shoulder: 22.9%
		Elbow/Forearm: 13.2%
		Wrist & Hand: 21.8%
		Thumb: 33.6%
Glover et al. [20]	68% Career Prevalence 68%	Shoulder: 14.8%
		Elbow/Forearm: 5.5%
		Wrist & Hand: 12.5%
		Thumb: 17.8%
McMahon et al. [21]	41% (1-Year Prevalence of Thumb WMSDs) Career Prevalence of 65%	Shoulder: ^a^
		Elbow/Forearm: ^a^
		Wrist & Hand: ^a^
		Thumb: 41%
Rossettini et al. [22]	49.3% (1-Year Prevalence of Thumb WMSDs) Career Prevalence of 70.8%	Shoulder: ^a^
		Elbow/Forearm: ^a^
		Wrist & Hand: ^a^
		Thumb: 49.3%
Rozenfeld et al. [23]	83% Career Prevalence of 80%	Shoulder: 42.2%
		Elbow/Forearm: 16%
		Wrist: 35.7%
		Thumb: 33.9%
West and Gardner [24]	55% Career Prevalence of 40%	Shoulder: 10%
		Elbow/Forearm: ^a^
		Wrist & Hand: 14%
		Thumb: ^ab^

^a^No data reported

^b^Thumb data included under hand data

### Risk factors for WRULDs

Table 4 illustrates potential risk factors in the physical therapy workplace, reported by PTs, that may contribute to or exacerbate the development of WRULDs. The most frequently reported risk factors included treating a large number of patients each day, performing manual therapy, continuing to work while injured, and working in the same position for long periods of time.

**Table 4:** Workplace Risk Factors

**Author**	**Treating Large # of Patients Per Day**	**Manual Therapy**	**Repetitive Tasks**	**Continuing to Work While Injured**	**Working At or Near Physical Limits**	**Working in Same Position for Long Time**	**Lack of Rest Breaks**	**Inadequate Training in Injury Prevention**
Adegoke et al. [13]	83.5%	67.8%	52.2%	52.2%%	46.9%	71.3%	61.7%	29.6%
Alnaser and Aljadi [14]	^b^	26%	5%	85%	^b^	10%	^b^	^b^
Buddhadev and Kotecha [16]	26.7%	^b^	^b^	1.7%	^b^	11.7%	8.3%	^b^
Campo et al. [17]	^b^	^a^	^a^	^b^	^b^	^b^	^b^	^b^
Campo et al. [18]	^b^	^a^	^a^	^b^	^b^	^b^	^b^	^a^
Chung et al. [19]	90.4%	72.0%	86.6%	77.7%	64.3%	73.2%	89.8%	42.7%
Cromie et al. [5]	41.4%	53.8%	52.3%	^a^	^a^	41.5%	^a^	3.1%
Glover et al. [20]	67%	49%	73%	52%	44%	67%	41%	14%
McMahon et al. [21]	76%^c^	70%^c^	86.0%^c^	69.0%^c^	56.0%^c^	^b^	49%^c^	40%^c^
Rossettini et al. [22]	^b^	68.5%^c^	^b^	66.7%^c^	^b^	64.8%^c^	^b^	^b^
Rozenfeld et al. [23]	62.4%	32.3%	58.1%	51.7%	35.5%	31.2%	^b^	12.9%
West and Gardner [24]	50%	50%	50%	51%	32%	58%	33%	6%

^a^specific percentages not reported

^b^no data reported

^c^specific to thumb disorders

Chung et al. [19] found that 90.4% of respondents identified “treating an excessive number of patients daily” as the most significant occupational risk factor. Further, 72.3% of respondents reported “working in a sustained position for an extended period of time” as a risk factor for developing shoulder disorders and 89.8% reported a “lack of rest breaks” as another contributing factor for developing WRULDs. Comparatively, Cromie et al. [5] reported that PTs who “treat a large number of patients in one day” and “take insufficient rest breaks during the day” were 3.2 times (OR 3.2, 95% CI 2.0-5.1) and 2.2 times (OR 2.2, 95% CI 1.4-3.8) more likely to experience work-related disorders of the wrist and hand, respectively. In a study by Adegoke et al. [13], similar findings showed that 83.5 % of PTs identified “treating a large number of patients per day” and 71.3% identified “working in the same position for long periods of time” as precipitating work-related risk factors for general WMSDs, an unspecified portion of which included WRULDs.

Cromie et al. [5] also discovered the “performance of manual orthopedic techniques” was associated with increased risk of elbow disorders (OR 3.5, 95% CI 1.9-6.7), wrist and hand disorders (OR 5.1, 95% CI 3.0-8.6), and thumb disorders (OR 5.5, 95% CI 3.5-8.6). Similarly, Campo et al. [18] concluded that PTs who performed manual therapy techniques, specifically soft tissue work, on more than 10 patients per day, had odds of developing wrist and hand WRULDs that were 13.61 times higher than those therapists who performed no soft tissue work (OR 13.61, 95% CI 2.91-63.78). Moreover, therapists who performed joint mobilization on more than 10 patients per day had odds of developing wrist WMSDs that were 7.95 times higher than PTs who did not perform joint mobilizations (OR 7.95, 95% CI 2.18-29.04) [18]. McMahon et al. [21] reported that 86% of PTs identified “repetitive tasks” and 70% identified “performing manual therapy techniques” as being major risk factors that contributed to their WRULDs, specifically of the thumb. Furthermore, PTs who spent between 31% and 60% of their time performing manual therapy were 3.4 times more likely to experience work-related thumb problems (OR 2.3 to 3.4, 95% CI 1.7-5.1) [21].

In nine of the 12 studies [5, 13, 14, 16, 19–24], PTs identified “continuing to work while injured” as a risk factor for the development of WRULDs. Specifically, authors reported 2.5 greater odds of PTs developing shoulder disorders (OR 2.5, 95% CI 1.5-4.2) [5] if working while injured and 66.7% of Italian PTs [22] identified this as an aggravating factor for thumb disorders. PTs in seven of the 12 studies [5, 13, 19–21, 23, 24] identified “working at or near physical limits” as a risk factor for the development of WRULDs. Finally, between 3.1% and 42.7% of PTs believed that “inadequate training in injury prevention” contributed to the development of WRULDs, with PTs in eight of the 12 studies citing this as a risk factor [5, 13, 18–21, 23, 24].

In addition to the workplace risk factors for WRULDs listed in Table 4, Glover et al. [20] noted other important risk factors. In their survey of PTs, respondents identified the following additional risk factors: “working in awkward or cramped conditions” (44%), “assisting patient during gait activities” (37%), and “working with confused or agitated patients” (25%). The PTs surveyed by Glover et al. [20] were also indicated whether they received a risk assessment in their current position. Fifty-six percent of PTs reported having a risk assessment in their current position, with 30% of PTs having regular, annual risk assessments [20]. Following these risk assessments, 74% stated that changes were made afterwards to reduce injury risk [20].

### Consequences of WRULDs

PT-reported consequences of WRULDs are listed in Fig. 2. Eight of the 12 studies [5, 13, 14, 18, 20, 21, 23, 24] found that very few PTs left the profession as a result of their WRULDs. Campo et al. [18] and McMahon et al. [21] reported low values (0.1% and 4%, respectively) of PTs leaving the profession due to WRULDs. Similar findings were reported by Rossettini et al. [22] that indicated 4.6% of Italian manual therapists changed careers. In addition, Glover et al. [20] found very few PTs surveyed retired early due to injury or left the profession entirely. As mentioned in all 12 studies [5, 13, 14, 16–24], PTs instead changed their work settings in response to their WRULDs, including areas other than direct patient care, such as academia or administration [5]. Although the injured body region was not specified, three sources indicated that between 11-39% of PTs changed their specialty area or left the physical therapy profession as a consequence of general WMSDs [5, 23, 24]. In response to work-related thumb disorders, McMahon et al. [21] reported that 19% of Australian PTs changed their area of practice.

In a specific study of orthopedic PTs by Campo et al. [17], PT respondents expressed concern over the possibility of their work-related wrist and hand pain limiting the longevity in their current work setting (47%) and affecting the longevity in their career as a PT (40%). A portion of the orthopedic PTs also identified a reduction in the quality of care provided (17%) and a reduction in job satisfaction (27%) as a consequence of their WRULDs [17].

In five of the 12 studies [5, 17, 22–24], WRULDs negatively impacted PTs’ performance of activities of daily living (ADLs), instrumental activities of daily living (IADLs), and recreational activities. In one such study by Rozenfeld et al. [23], the authors found that 15.8% of PTs with shoulder disorders, 7.4% with elbow disorders, 7.5% with wrist and hand disorders, and 8.3% with thumb disorders experienced disruptions in performing their ADLs, IADLs, and leisure activities due to their WRULDs. In contrast, Cromie et al. [5] reported slightly lower values, with 6.9% of PTs with shoulder disorders, 5% with elbow disorders, 6.5% with wrist and hand disorders, and 5.4% with thumb disorders experiencing difficulty with completing ADLs, IADLs, and recreational activities as a consequence of their WRULDs. Among Italian manual therapists with work-related thumb disorders [22], 9.3% experienced impairment with performing ADLs and IADLs, and 2.8% required a temporary suspension of recreational sports.

In addition to the prevalence of WMSDs, a few authors investigated if PTs sought medical care in response to their work-related injuries. West and Gardner [24] found that 45% of Australian orthopedic PTs with work-related hand disorders consulted with a physician. Meanwhile, Rozenfeld et al. [23] found that 15.8% of PTs with shoulder disorders, 14.8% with elbow disorders, 7.5% with wrist and hand disorders, and 2.8% of PTs with thumb disorders sought consultation with a physician regarding their WRULDs. Campo et al. [18] reported that only 2.8% of PTs with shoulder disorders, 1.4% with elbow disorders, and 3.6% with wrist and hand disorders visited a physician due to their WRULDs.

Finally, authors reported on the consequences of lost time due to a work-related injury. Specifically, Rozenfeld et al. [23] reported that 5.3% of PTs with shoulder disorders, 7.4% with elbow disorders, 15.0% with wrist and hand disorders, and 13.9% with thumb disorders were absent from work as a result of their WRULDs. In contrast, Campo et al. [18] found that only 1% of PTs with shoulder disorders, 0.2% with elbow disorders, and 1.6% with wrist and hand disorders lost time at work due to their WRULDs. These low rates reported by Campo et al. [18] were similar to those found by Cromie et al. [5], who reported a combined total of 6.7% of PTs were prevented from working due to their respective work-related shoulder, elbow, wrist and hand, and thumb disorders. In another study investigating lost time among Italian manual therapists [22], 5.6% of PTs experienced temporary suspension from work due to their work-related thumb disorders.

### Coping strategies for WRULDs treatment and prevention

Frequently reported coping strategies utilized by the PTs, either as an intervention in response to upper-limb injury or as a preventative measure used against future risk of injury, are presented in Fig. 3. Two categories of coping strategies proposed by Cromie et al. [5] included reactive and preventative strategies. Reactive strategies are implemented “in response to injury or perceived risk of injury” and consist of PTs employing self-protective behaviors, such as using another body part to administer a manual technique or ceasing the use of techniques that provoke WRULD symptoms [5]. Preventative strategies are put into practice to “reduce the risk of injury” and include the PTs modifying and altering the treatment environment, such as adjusting plinth height [5].

Of the two proposed coping strategies, reactive strategies were more frequently reported, with 11 of the 12 studies [5, 13, 14, 16–20, 22–24] centered on treatment technique modifications and alterations in body mechanics. For example, Rossettini et al. [22] found the most common coping strategies among Italian manual PTs with work-related thumb disorders included various reactive treatment modifications. Respondents in this study reported modifications of “altering practice positions” (69.5%), “changing working positions frequently” (57.4%), and “incorporating breaks into work schedules” (27.8%) [22]. West and Gardner [24] found that 91% of Australian PTs with wrist and hand disorders modified their PT techniques and 77% sought physical therapy treatment in response to their WRULDs. Similarly, Rozenfeld et al. [23] determined that 30% of PTs with wrist disorders and 41.7% with thumb disorders sought treatment, either treating themselves or receiving treatment from colleagues, while only 10.5% with shoulder disorders and 7.4% with elbow disorders pursued treatment. A study of orthopedic PTs by Campo et al. [18] reported that 52% of PTs altered their techniques and body mechanics when performing hands-on interventions (e.g., changing hand placement, alternating upper limb sides, or using the elbow instead of the thumb during soft tissue mobilizations). Similar strategies were undertaken by PTs surveyed by Glover et al. [20], with a majority of PTs reportedly making adjustments to plinth height (86%) or modifying their or the patient’s position (79%).

Campo et al. [18] concluded a smaller percentage of PTs utilized forms of preventative strategies. The preventative strategies used included applying braces, splints, tools, and other assisted devices (27%); being more selective in their use of manual therapy techniques (21%); and incorporating therapeutic exercise such as strengthening, stretching, and conditioning for rehabilitation or prevention purposes (13%) [17]. Comparably, West and Gardner [24] reported that 55% of PTs utilized equipment during manual therapy such as a splint or brace to reduce future excess force on their hands and wrists.

Further underscoring the use of and need for preventative strategies, Rozenfeld et al. [23] suggested the implementation of ongoing in-service training programs for practicing PTs that focus on WRULDs prevention and intervention. Moreover, 16% of PTs in a study by Campo et al. [17] identified the importance of and the need for early provision of education and preventative training in physical therapy programs to heighten awareness of WRULDs and to mitigate future development of WRULDs.

## Discussion

The objectives of this systematic review were to examine the 1-year prevalence of WRULDs among PTs, identify the associated risk factors in the physical therapy workplace, assess the consequences of WRULDs, and determine the coping strategies utilized by PTs to prevent or manage WRULDs.

The 1-year WRULDs prevalence rates varied widely across the 12 studies [5, 13, 14, 16–24]. Authors concluded that anywhere from 28% to 92.4% of PTs were affected annually by general WMSDs. Among WRULDs, all 12 studies [5, 13, 14, 16–24] reported prevalence rates of thumb disorders that ranged from 7.6% to 52.5%. Further, 10 of the 12 studies [5, 13, 14, 16–20, 23, 24] indicated the prevalence of wrist and hand disorders spanned a wide range from 5% to 66.2%. Nine studies [5, 13, 14, 16, 18–20, 23, 24] stated the 1-year prevalence of shoulder disorders ranged from 3.2% to 45.2% of PTs. Finally, the prevalence of elbow injuries was described in only seven of the 12 studies [5, 13, 16, 18–20, 23] and prevalence rates fell within a narrower range of 1.4% to 16%.

Many factors may have contributed to these disparate prevalence results. First, the components of stoicism and reticence within the culture of physical therapy may have contributed to the underreporting rates of WRULDs, viewed by PTs as inherent to physical therapy work [8]. A second contributing factor may be due to the variations in studies’ methods and materials. Since some of the survey tools utilized were designed by the studies’ authors, the tools’ reliability and validity are unknown, which may impact the reporting of their results. However, other studies employed more standardized survey tools like the Nordic Musculoskeletal Questionnaire [24], possibly improving the reliability and validity of their results. Furthermore, self-report questionnaires may be impacted by respondents’ recall bias, with PTs potentially overreporting or underreporting their perceived experience of WRULDs [22].

Further, prevalence rate disparities may have also been impacted by the studies’ differing definitions of quantifiable criteria for WRULDs classification. Some articles used more general definitions rather than strict definitions of what qualified as a reportable WRULD. Ten of the included studies [5, 13, 14, 16, 19–24] used a broad and nonspecific definition of work-related disorders, such as the one described by West and Gardner [24] that defined the disorders as “pain or discomfort lasting more than 3 days that the respondent felt was caused by their work as a PT.” However, the remaining two studies [17, 18] employed a narrower definition described by Campo et al. [17] and included a specific time and pain intensity rating scale. Competing and incongruous definitions used across the studies create challenges in making direct and accurate comparisons amongst the prevalence data.

Other key factors potentially influencing prevalence rates relate to the variations in PT practice setting and geographic location. For instance, the respondent pools of Campo et al. [17] and Rossettini et al. [22] were specifically characterized as orthopedic specialists and manual therapy specialists whereas the PT population included in the study by West and Gardner [24] was not well described. Other dissimilarities may be skewed by geography and culture. Nine different geographic locations where these 12 studies were conducted may have had variations in certain aspects of physical therapy education, training, and practice [5, 13, 14, 16–24]. Finally, organizational system difference in practice settings and geographic locations may also influence prevalence rates. Such differences impacting prevalence rates may include the work-pace, productivity, total days of annual leave, continuing education requirements, quality of care requirements, and reimbursement models.

Risk factors for the development of WRULDs reported by most studies included treating a high volume of patients per day, frequently performing manual therapy techniques, working while injured, and working for long periods of time in sustained positions [5, 13, 14, 16–24]. These workplace risk factors place inordinate forces on the body that often lead to a biomechanical overload of the upper limb. These findings are similar to risk factors cited by studies of WRULDs in occupational therapists (OTs) [26, 27]. Moreover, among the PT and OT respondents in one study, a majority of injuries with manual therapy occurred to the wrist and hand while injuries associated with transfers and lifts primarily occurred in the shoulders or elbows [26]. Although PTs and OTs had similar 1-year prevalence rates for WRULDs in this study [26], the prevalence of elbow, wrist, and hand disorders were significantly less in OTs when compared to PT respondents in the studies included in this review [5, 13, 14, 17, 19, 21–23]. Thus, PTs may be more susceptible to experiencing WRULDs since they are exposed daily to more than one risk factor [5], specifically related to the frequent usage of their wrists and hands to perform manual therapy techniques. These findings underscore the need for effective preventative strategies to reduce the risk of therapists experiencing WRULDs while performing routine patient care activies.

Some other potential risk factors for the development of WRULDs included gender, age, professional years of experience, work setting, and genetic factors such as anthropometric differences or ligamentous stability [17, 21]. These elements were not considered in this review due to disagreement in the literature as to whether the factors may serve as protective factors against or risk factors for the development of WRULDs among PTs [5, 13, 14, 16–24].

The results of this review indicated that the consequences of WRULDs vary from activity limitations to leaving the profession entirely. Cromie et al. [5] reported that nearly 1 in 6 PTs changed their work setting or left the physical therapy profession in response to general work-related injuries and disorders, a portion of which involved the upper limb. WRULDs may also lead to interference with PTs’ performance of ADLs, IADLs, and leisure activities [5, 17, 22–24]. WRULDs severe enough may require a consultation with a physician, resulting in work time lost [5, 18, 22–24]. Another consequence of WRULDs can be a diminishment of PTs’ longevity in active physical therapy practice [17].

Interestingly, there appeared to be an incongruence between risk factors for WRULDs identified in the studies and the strategies reportedly used by PTs to address these risk factors. While treating a large number of patients per day and continuing to work while injured were commonly cited by PTs as workplace risk factors [5, 13, 14, 16, 19–24], these risks were not addressed by the reported coping strategies to treat or prevent WRULDs. This discrepancy suggests the need for more holistic and systemic strategies within the physical therapy profession to mitigate these risk factors for WRULDs. Identifying effective risk-reducing strategies is a prime area for further investigation, but could include changes in productivity standards or the culture of stoicism within the physical therapy profession.

Our findings of the contrasting 1-year prevalence rates of WRULDs among PTs are similar to those rates reported in the literature for other healthcare professions. In their systematic review of allied health professionals, Anderson and Oakman [28] found a 1-year prevalence of WMSDs ranged between 28% and 96%. Although they included literature on multiple allied health professions, they reported similar risk factors associated with predominantly physical work-related tasks. The authors also reported that performing manual therapy tasks was associated with higher rates of pain or discomfort in the fingers and hands. Any discrepancies in risk factors and consequences reported by Anderson and Oakman [28] and this present systematic review is likely due to their inclusion of spine and lower extremity WMSDs.

In a second systematic review, Vieira et al. [29] reported a career prevalence of thumb disorders of 57% to 83% in PTs who performed manual therapy techniques. The authors cited additional risk factors for WRULDs, including treating a high volume of patients per day and performing repetitive work. Consistent with our findings, the most common consequences and coping strategies identified in their review were mostly reactive, such as modifying treatment techniques or changing practice setting [29]. When comparing prevalence rates of PTs to perioperative nurses, a systematic review of 22 studies reported rates that were comparable to that of PTs for shoulder and elbow WRULDs [30]. Specifically, perioperative nurses’ highest 1-year prevalence of shoulder WMSDs was 44%, whereas elbow WMSDs was 18% [30]. These findings are similar to the highest reported rates among PTs of 45.2% and 16% within the shoulder and elbow body regions, respectively [19, 23]. Wrist and hand disorders, however, showed significantly different prevalence rates among perioperative nurses and PTs. The highest-reported 1-year prevalence of wrist and hand disorders among perioperative nurses was 29% [30]. In contrast, the highest reported rate among PTs was 66.2% [17]. Some of the similarities in shoulder and elbow WRULDs may be related to the physically intensive nature of both professions. Moreover, perioperative nurses and PTs encounter similar work-related risk factors during their workdays such as sustained body positions and postures, repetitive and forceful tasks, high patient volume, and lack of rest breaks. However, the differences in wrist and hand WRULDs likely exist due to the frequent usage of manual therapy techniques by PTs during their workday. In line with the findings of this present systematic review on WRULDs among PTs, Clari et al. [30] identified the need for implementation of “environmental, ergonomic, and organizational factors” to reduce WRULDs among perioperative nurses. These findings and corresponding recommendations support the findings of this study and provide insight into the widespread impact that WRULDs have on physically demanding professions.

### Limitations

One limitation to our study is a lack of methodological standardization across the 12 studies reviewed. Studies included in our analysis used different outcome measures and surveys, which can be subject to recall bias, and the participants varied across PT practice settings and geographic regions. One strategy we employed to minimize recall bias was to limit the review of prevalence data to one year. Further, studies in our review had inconsistent definitions of WRULDs, potentially impacting the varying rates reported by PTs. Another limitation of this study is publication bias, most notably the requirement for publication in the English language and in peer-reviewed journals. Additionally, previous literature noted the combination of both biomechanical and psychosocial factors that may contribute to WRULDs [31]. However, this study did not evaluate the psychosocial risk factors, such as low social support at work, that may have contributed to the the prevalence rates or coping strategies to reduce WRULDs. Finally, due to the disagreement in the literature, we excluded the consideration of gender, age, professional years of experience, work setting, and genetic factors to the development of WRULDs. Despite these limitations, we are confident that these limitations did not compromise or significantly alter the conclusions of this systematic review. Future studies should employ valid and reliable measurement tools and standardized definitions to allow for meaningful comparisons and recommendations on this important topic.

### Clinical implications

The findings of this systematic review suggest that WRULDs are inevitable in the practice of physical therapy. Educating PTs on the existing threat of WRULDs and the presence of workplace risk factors is crucial to the successful reduction of WRULDs. In the majority of studies, PTs adopted reactive strategies more frequently than preventative strategies [5, 13, 14, 16–19, 22–24]. Both of these coping strategy categories are efficacious and must be acknowledged and applied by PTs given the inevitability of WRULDs in the practice of physical therapy. Vital to the reduction of WRULDs among PTs is the need for a fundamental shift to a more proactive mindset and the implementation of preventative coping strategies to promote longevity in the practice of physical therapy.

## Conclusion

Due to the hands-on, physically intensive nature of the physical therapy profession, the 1-year prevalence of WRULDs among PTs remains persistent and continues to rise. Workplace risk factors that substantially increase the risk of developing WRULDs have been well identified in the literature. WRULDs can negatively impact PTs’ work performance and lifestyle and can ultimately compromise PTs’ work longevity. Essential to the successful reduction of WRULDs is the amplified education and heightened cognizance of the existing threat of WRULDs in physical therapist practice. In addition to education, holistic and systemic strategies are needed to address workplace risk factors, mitigate negative consequences, and institutionalize effective coping strategies for WRULDs. The conclusions from this systematic review are actionable and demand continued research on the topic of WRULDs among PTs to protect, preserve, and prolong the use of their upper limbs.

## Data Availability

All data generated or analyzed during this study are included in this published article [and its supplementary information files].

## Appendix

Table 5

**Table 5:** JBI critical appraisal checklist for studies reporting prevalence data

1. Was the sample frame appropriate to address the target population?
2. Were study participants sampled in an appropriate way?
3. Was the sample size adequate?
4. Were the study subjects and the setting described in detail?
5. Was the data analysis conducted with sufficient coverage of the identified sample?
6. Were valid methods used for the identification of the condition?
7. Was the condition measured in a standard, reliable way for all participants?
8. Was there appropriate statistical analysis?
9. Was the response rate adequate, and if not, was the low response rate managed appropriately?

## Abbreviations

WMSDs: Work-related musculoskeletal disorders
PTs: Physical therapists
WRULDs: Work-related upper limb disorders
PRISMA: Preferred Reporting Items for Systematic Reviews and Meta-Analyses
MeSH: Medical Subject Headings
JBI: Joanna Briggs Institute
NMQ: Nordic Musculoskeletal Questionnaire
ADLs: Activities of daily living
IADLs: Instrumental activities of daily living
